# PLA2G2A across tumor compartments and its relevance to drug resistance

**DOI:** 10.20517/cdr.2026.90

**Published:** 2026-10-28

**Authors:** Kexun Li, Zilong Qian, Jianzhe Zhang, Jie Mao, Chengwei Bi, Simiao Lu, Xin Tang, Jin Liu, Yongtao Han, Xuefeng Leng

**Affiliations:** ^1^Department of Thoracic Surgery, Sichuan Clinical Research Center for Cancer, Sichuan Cancer Hospital & Institute, Sichuan Cancer Center, Affiliated Cancer Hospital of University of Electronic Science and Technology of China (Sichuan Cancer Hospital), Chengdu 610041, Sichuan, China.; ^2^Department of Thoracic Surgery I, Key Laboratory of Lung Cancer of Yunnan Province, Yunnan Cancer Hospital, The Third Affiliated Hospital of Kunming Medical University (Yunnan Cancer Hospital), Kunming 650118, Yunnan, China.; ^3^Department of Urologic Surgery, Key Laboratory of Lung Cancer of Yunnan Province, Yunnan Cancer Hospital, The Third Affiliated Hospital of Kunming Medical University (Yunnan Cancer Hospital), Kunming 650118, Yunnan, China.; ^4^School of Public Health, Chongqing Medical University, Chongqing 400016, China.; ^#^These authors contributed equally to this work and share first authorship.

**Keywords:** PLA2G2A, cancer drug resistance, tumor microenvironment, cancer-associated fibroblasts, lipid mediators, immunotherapy resistance, extracellular vesicles

## Abstract

Drug resistance in cancer is shaped not only by tumor-cell-intrinsic mutations but also by spatially organized microenvironmental states. PLA2G2A, encoding group IIA secreted phospholipase A2 (sPLA2-IIA), is usually discussed as an inflammatory lipid enzyme; however, its cancer relevance depends strongly on cellular source, localization and disease stage. This Review uses a compartment-resolved framework that annotates four domains - source, spatial niche, linked biochemical output and functional or therapy-response phenotype - and separates PLA2G2A-specific mechanistic evidence from evidence for therapy or immune resistance. Several cancer studies provide PLA2G2A-specific functional evidence for invasion, proliferation/stemness, non-catalytic signaling or T-cell dysfunction, but direct demonstration that PLA2G2A changes response to a defined anticancer therapy remains sparse. The pancreatic ductal adenocarcinoma cancer-associated fibroblast study provides the strongest resistance-proximal immune-functional evidence, whereas mucinous colorectal extracellular vesicles, ovarian ascites, invasive-front fibroblasts, treatment-remodeled stroma and prognostic signatures require more direct therapy-response validation. Gastric and intestinal epithelial data further caution that PLA2G2A may accompany differentiation, invasion restraint, antimicrobial defense or barrier protection. We therefore argue against treating PLA2G2A as a universal oncogenic target. Instead, it should be advanced as a mechanism-matched biomarker or therapeutic hypothesis only when compartment, biochemical-output, functional and perturbational evidence converge.

## INTRODUCTION

Drug resistance and immune escape are shaped by tumor-cell-intrinsic and tumor microenvironment (TME) alterations. Secreted phospholipases A2 (PLA2) are extracellular lipid-modifying enzymes with an established catalytic mechanism for phospholipid hydrolysis^[1]^. Their roles in inflammatory signaling are also established, and family-level evidence has linked sPLA2-mediated signaling with cancer-related bioprocesses^[2,3]^. They are particularly relevant to microenvironmental interpretation as their bioeffects may depend on where they are produced, retained, and functionally coupled to neighboring cells.

PLA2G2A encodes group IIA secreted phospholipase A2 (sPLA2-IIA), a small-sized enzyme that hydrolyzes the sn-2 ester bond of membrane phospholipids, generating free fatty acids and lysophospholipids. By releasing arachidonic acid (AA), PLA2G2A can feed the cyclooxygenase (COX), lipoxygenase (LOX), and oxylipin pathways^[1-3]^. This biochemical pathway position places PLA2G2A upstream of inflammatory lipid networks that can influence dendritic cells, macrophages, fibroblasts, epithelial stress, and T-cell fitness.

Despite such biochemical potential, cancer data do not support a simple interpretation of PLA2G2A being uniformly tumor-promoting or protective. In differentiated gastric and intestinal epithelial settings, PLA2G2A can be involved in invasion restraint, antimicrobial defense, or barrier integrity. In contrast, in desmoplastic, mucin-rich, ascites-rich, adipose-associated, or therapy-remodeled niches, it may mark lipid processing, extracellular matrix (ECM) retention, immune suppression, or resistance-associated remodeling. The central challenge is therefore not whether PLA2G2A is expressed, but in which compartment it is produced, where the protein accumulates, what lipid products are generated, and whether immune or stromal function changes accordingly.

This review is designed for therapy-oriented interpretation. We evaluate PLA2G2A as a conditional node in cancer drug resistance rather than as a pan-cancer oncogene. We prioritize evidence that connects PLA2G2A to drug-response biology, immune escape, stromal vulnerability, or biomarker development, while explicitly separating causal cancer biology from direct treatment-resistance evidence. The proposed framework distinguishes PLA2G2A-dependent mechanisms from PLA2G2A-associated biomarkers and broader inflammatory bystander states. A schematic overview of PLA2G2A-related signaling pathways and their context-dependent biological effects is provided in the Visual Abstract.

## SCOPE, TERMINOLOGY AND EVIDENCE GRADING

A family-wide synthesis of secreted PLA2 isoforms is provided, emphasizing enzymatic and ligand-like signaling, epithelial-mesenchymal transition (EMT), angiogenesis, immunomodulation, and inhibitor development^[3]^. The present review does not claim to be the first to address the role of PLA2G2A in cancer. Its narrower contribution is fourfold: (1) PLA2G2A-specific annotation of cell source and spatial niche rather than family-level cataloging; (2) an operational four-domain source-niche-biochemical-output-function framework; (3) separate grading of PLA2G2A-specific mechanistic causality and therapy-resistance directness; and (4) treatment-oriented, falsifiable predictions, and validation requirements. These additions complement rather than supersede the broader synthesis of evidence^[3]^.

This article is a narrative mechanistic synthesis rather than a systematic review or meta-analysis. The literature was searched in the PubMed, Web of Science, and Google Scholar databases from inception to June 30, 2026 using combinations of the terms: “PLA2G2A”, sPLA2-IIA, secreted phospholipase A2, cancer, TME, cancer-associated fibroblast (CAF), CD8, macrophage, extracellular vesicle (EV), AA, eicosanoid, adipocyte, obesity, microbiota, immunotherapy, and therapy resistance. Studies on PLA2G2A expression, localization, perturbation, secretome, or EV profiling, lipid readouts, immune endpoints, clinical outcome, or therapy-response association were included. Noncancer studies were used only when they clarified catalytic, receptor, matrix, vascular, adipose, microbial, or barrier mechanisms that shape the interpretation of disease pathogenesis. Purely *in silico* signature studies were retained as mechanistic-level M-C evidence; non-peer-reviewed abstracts, reports without isoform-resolved PLA2G2A data, and family-level sPLA2 observations that could not resolve PLA2G2A were excluded from PLA2G2A-specific mechanistic claims.

We used the term “PLA2G2A-defined tumor ecosystem” conservatively. Four annotation domains were considered: (i) an identified cellular or extracellular source; (ii) spatial or compartmental localization, such as epithelium, stroma, invasive front, ascites, or EVs; (iii) a linked biochemical output in the same specimen or model, such as AA/prostaglandin E2 (PGE2)/oxylipins, lysophospholipids, receptor-proximal signaling or matrix engagement; and (iv) a linked functional phenotype, including immune or stromal function, invasion, tumor growth or treatment response. A lipid mechanism is invoked only when PLA2G2A is linked to quantified lipid products rather than inferred from expression alone. Studies lacking one or more domains remain informative but are described by those actually determined rather than treated as fully PLA2G2A-defined ecosystems.

We developed a review-specific, dual-axis evidence-grading framework to distinguish PLA2G2A-specific mechanistic causality from therapy-resistance directness [Table 1]. This framework was designed specifically for the present narrative review and was not adapted from an existing standardized evidence-grading system. Mechanistic specificity was graded M-A to M-D: M-A requires PLA2G2A-specific genetic gain or loss, neutralization or pharmacologic inhibition, or controlled exposure to purified/recombinant PLA2G2A together with a functional readout; M-B denotes compartment-resolved or cell-state-related functional association without PLA2G2A-specific perturbation; M-C implies the association of bulk clinical, prognostic, or multi-gene signatures; and M-D signifies inference from adjacent noncancer or non-PLA2G2A models. Resistance directness was scored separately as R3-R0: R3 requires a defined experimental anticancer therapy or clinical response linked to PLA2G2A-specific manipulation; R2 denotes resistance-proximal immune or survival dysfunction linked directly to PLA2G2A without a defined therapy-response test; R1 indicates the association of treatment-stage, outcome, or predicted responses without PLA2G2A-specific response manipulation; and R0 implies no direct evidence of therapy resistance. Under this framework, several studies reached M-A for cancer biology, whereas the pancreatic ductal adenocarcinoma (PDAC) CAF report was M-A/R2, and no PLA2G2A-specific cancer report in the evidence map reached R3. These labels rank the proof supporting this specific claim under discussion rather than overall study quality.

**Table 1:** Review-specific dual-axis evidence framework separating PLA2G2A-specific mechanistic causality from therapy-response directness

**Axis**	**Level**	**Minimum criterion**	**Interpretation in this review**
Mechanistic specificity	M-A	PLA2G2A-specific genetic gain/loss, neutralization, selective pharmacologic inhibition, or controlled purified/recombinant PLA2G2A exposure plus a functional readout	Supports PLA2G2A-specific causal inference for the stated phenotype; does not by itself establish therapy resistance
Mechanistic specificity	M-B	Compartment-resolved source/niche association with linked functional, spatial, EV/secretome, lipidomic or cell-state evidence but without PLA2G2A-specific perturbation	Supports a source- or niche-specific mechanistic hypothesis; causality remains incomplete
Mechanistic specificity	M-C	Bulk RNA, prognostic model, immune/lipid signature, survival association or predicted treatment-response association	Useful for stratification or hypothesis generation; insufficient for mechanism
Mechanistic specificity	M-D	Inference from adjacent non-cancer models or from other sPLA2 isoforms	Hypothesis-generating only
Resistance directness	R3	A defined anticancer drug, radiotherapy, or immunotherapy response is altered by PLA2G2A-specific manipulation in an experimental model or clinical setting	Direct therapy-resistance evidence
Resistance directness	R2	PLA2G2A-specific manipulation/exposure changes a resistance-proximal immune or survival phenotype, but no defined anticancer therapy-response experiment is performed	Mechanistically relevant to resistance but not direct treatment-response evidence
Resistance directness	R1	Treatment-stage, clinical outcome, or measured or predicted treatment-response association, including prospective source-resolved clinical associations, without PLA2G2A-specific response manipulation	Associative therapy relevance only
Resistance directness	R0	No direct treatment-response or resistance endpoint	Cancer-biology evidence only; do not relabel as resistance evidence

This evidence-grading framework was developed specifically for the present narrative review and was not adapted from an established standardized evaluation system. The grades represent the strength of PLA2G2A-specific mechanistic evidence and the directness of therapy-resistance evidence, rather than the overall methodological quality of individual studies. M: Mechanistic specificity; R: resistance directness; EV: extracellular vesicle; sPLA2: secreted phospholipase A2.

Four testable predictions follow from this framework. First, in PLA2G2A-positive CAF-enriched PDAC, spatial proximity between PLA2G2A-positive CAFs and CD8^+^ T cells must be associated with reduced interferon gamma (IFNG), tumor necrosis factor (TNF), granzyme B (GZMB), CD69, and OX40 independently of bulk CD8^+^ T-cell abundance. Second, in mucinous or EV-active colorectal cancer (CRC), the prespecified primary endpoint should be a PLA2G2A-linked lipid-output score ascertained in EV-rich samples; for example: AA, PGE2, and selected oxylipins. The primary endpoint’s relation with EV-associated PLA2G2A has to be tested with a prespecified multivariable regression model adjusted for tumor burden and BRAF status, with bulk tumor PLA2G2A evaluated via a comparator model. Mucin abundance and focal-adhesion/VEGF pathway scores are to be treated as secondary exploratory endpoints. Such a prediction would be considered unsupported if EV-associated PLA2G2A does not show any independent association with the lipid-output score or if the bulk tumor PLA2G2A-based comparator model performs equally well or better. Third, in gastric or intestinal epithelial tumors, PLA2G2A co-occurrence with differentiation, Paneth-cell, goblet-cell, or barrier markers must predict nontumor-promoting or protective biology unless stromal, EV, or lipid-niche data indicate otherwise. Fourth, longitudinal sampling should determine whether PLA2G2A marks a transient therapy-remodeling window or a stable resistance state.

## MOLECULAR BASIS OF PLA2G2A CONTEXT DEPENDENCE

### Dual catalytic and non-catalytic biology

PLA2G2A context dependence begins with its dual molecular identity. As an enzyme, sPLA2-IIA hydrolyzes phospholipids and increases local availability of AA and lysophospholipids^[1-3]^. These products can feed prostaglandin, leukotriene and oxylipin pathways. In drug-resistant tumors, this catalytic output may amplify inflammatory survival signaling, stromal activation, myeloid skewing or T-cell dysfunction. The relevant therapeutic implication is that PLA2G2A expression alone cannot predict whether direct enzyme inhibition, downstream COX/PGE2 blockade, LOX-axis targeting or stromal-state modulation will be most rational.

Several studies show that sPLA2-IIA also has signaling outputs that cannot be reduced to catalysis. Exogenous sPLA2-IIA, including catalytically inactive variants, can promote PLA2G2A promoter activity through the M-type receptor and heparan-sulfate proteoglycans (HSPGs), creating autocrine or paracrine amplification loops^[4,5]^. Lung and prostate cancer models link sPLA2-IIA to HER/ERBB-driven stem-like phenotypes^[6]^. Direct integrin binding has been demonstrated for alpha-v beta-3, alpha-4 beta-1 and alpha-5 beta-1 in biochemical and inflammatory-cell systems^[7]^, while vimentin-associated trafficking suggests additional non-catalytic signaling routes^[8]^. More recently, structure-informed cyclic peptides targeting human group IIA secreted phospholipase A_2_ (hGIIA)-epidermal growth factor receptor (EGFR) and hGIIA-vimentin interactions provided preclinical proof-of-concept that protein-protein interactions can be pharmacologically separated from catalytic inhibition in prostate cancer models^[9]^. We did not identify a cancer study that cleanly compares wild-type and catalytically inactive PLA2G2A while holding cell source, localization and receptor-binding context constant.

### Transcriptional integration and autocrine reinforcement

PLA2G2A expression integrates inflammatory, differentiation, endocrine, and metabolic inputs. Inflammatory regulation of PLA2G2A is context-dependent: elements of C/EBP, NF-κB, and Ets promoters participate in autocrine control in vascular smooth-muscle models^[4]^. However, IFN-γ-induced expression has been linked to STAT3 activation and is further modulated by the surrounding cytokine milieu^[10]^. NFATc2-containing circuitry extends induction downstream of C/EBP in hepatocyte models^[11]^. Conversely, thyroid hormone receptor β can repress PLA2G2A transcription, and PPAR-family effects are model-dependent rather than uniformly activating or inhibitory^[5,12,13]^. Thus, PLA2G2A is often a readout of the tissue state rather than an isolated driver.

The ability of sPLA2-IIA to reinforce its own expression through the M-type receptor, HSPG, and cPLA2-linked circuitry is therapeutically relevant^[4,5]^. PPARα participates in an autocrine loop in mesangial-cell models^[5]^, whereas other PPAR-family contexts can repress PLA2G2A^[12]^; these observations must not be condensed into a universal PPAR → PLA2G2A directional relationship. Resistance-associated ecosystems commonly require sustained low-grade signaling rather than transient inflammatory spikes. A locally retained secreted enzyme that can reinforce its persistence may help stabilize a resistant niche, but such a possibility requires compartment-specific validation in cancer models.

### Matrix retention and receptor-proximal signaling

Where PLA2G2A is retained may be more important than how much is expressed. In atherosclerotic lesions, group IIA phospholipase A2 colocalizes with decorin, and decorin promotes collagen binding and enhances enzymatic activity^[14]^. A similar principle may apply to ECM-rich cancers: desmoplastic stroma, invasive fronts and peritumoral adipose tissue can convert a secreted molecule into a spatially restricted signaling reservoir. Bulk RNA or serum protein levels may therefore underrepresent biologically active matrix-retained PLA2G2A.

The downstream map remains incomplete. EGFR/HER-family associations, HSPG-dependent amplification, direct integrin binding and vimentin-associated localization suggest candidate links to adhesion, receptor clustering, internalization, mitogen-activated protein kinase (MAPK), PI3K-AKT, FAK/Src and NF-κB programs^[6-9,14]^. These should be presented as testable axes rather than as established pan-cancer mechanisms. The distinction is critical because a negative result with a catalytic inhibitor would not exclude non-catalytic PLA2G2A biology [Figure 1].

**Figure 1:**
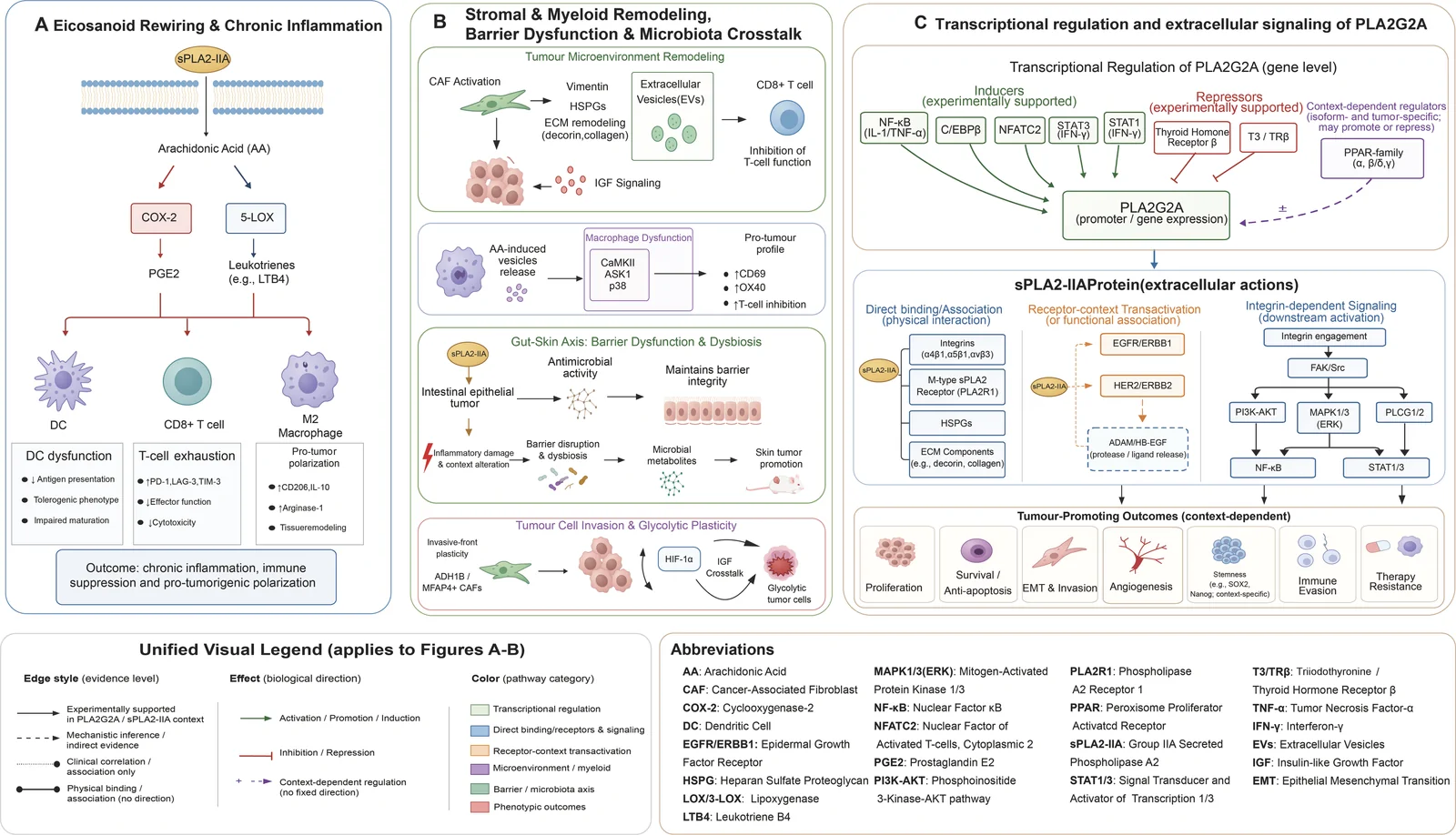
(A) Eicosanoid rewiring and chronic inflammation; (B) Stromal and myeloid remodeling, barrier dysfunction and microbiota crosstalk; (C) Context-dependent PLA2G2A signaling circuitry. sPLA2-IIA: Group IIA secreted phospholipase A2; AA: arachidonic acid; COX-2: cyclooxygenase-2; LOX: lipoxygenase; PGE2: prostaglandin E2; LTB4: leukotriene B4; DC: dendritic cell; PD-1: programmed cell death protein 1; LAG-3: lymphocyte-activation gene 3; TIM-3: T-cell immunoglobulin and mucin domain-containing protein 3; IL-10: interleukin-10; CAF: cancer-associated fibroblast; HSPGs: heparan sulphate proteoglycans; ECM: extracellular matrix; EV: extracellular vesicle; IGF: insulin-like growth factor; CaMKII: calcium/calmodulin-dependent protein kinase II; ASK1: apoptosis signal-regulating kinase 1; ADH1B: alcohol dehydrogenase 1B; MFAP4: microfibril-associated protein 4; HIF-1α: hypoxia-inducible factor 1α; NF-κB: nuclear factor κB; C/EBPβ: CCAAT/enhancer-binding protein β; NFATC2: nuclear factor of activated T cells, cytoplasmic 2; STAT: signal transducer and activator of transcription; IFN-γ: interferon-gamma; T3: triiodothyronine; TRβ: thyroid hormone receptor β; PPAR: peroxisome proliferator-activated receptor; PLA2R1: phospholipase A2 receptor 1; EGFR: epidermal growth factor receptor; ERBB: ErbB family of receptor tyrosine kinases; HER: human epidermal growth factor receptor; ADAM: a disintegrin and metalloproteinase; HB-EGF: heparin-binding epidermal growth factor-like growth factor; FAK: focal adhesion kinase; PI3K-AKT: phosphoinositide 3-kinase-AKT pathway; MAPK: mitogen-activated protein kinase; ERK: extracellular signal-regulated kinase; PLCG: phospholipase C γ; EMT: epithelial-mesenchymal transition.

### Contexts in which progression and therapy-response phenotypes can diverge

A major limitation of pan-cancer biomarker interpretation is the assumption that high PLA2G2A is uniformly adverse. Gastric cancer illustrates the opposite possibility. PLA2G2A was identified as a β-catenin/TCF target that suppresses invasion and metastasis in experimental models^[15]^, and clinical studies associated its expression with favorable histologic features, reduced metastasis and prolonged survival^[16,17]^. These findings support a differentiation-associated epithelial program, although they do not prove independent causal protection after adjustment for tumor grade, stage, histologic subtype and immune infiltration.

Intestinal models add a barrier-protective configuration. PLA2G2A can suppress tumorigenesis in Muc2-deficient and azoxymethane-treated mice and is associated with goblet-cell expression and mucus- and microbial-defense-related programs^[18,19]^. In cholangiocarcinoma, epigenetic repression of PLA2G2A can accompany progression in some contexts^[20]^. These examples argue that direct PLA2G2A blockade could be undesirable when the dominant biology is epithelial differentiation, antimicrobial defense or barrier maintenance.

### Position within the secreted PLA2 family

PLA2G2A has to be interpreted within a broader secreted phospholipase A2 family, but comparator isoforms are to be judged based on the same source-niche-output-function questions rather than by looser narrative criteria^[3]^. In ovarian cancer, PLA2G3 provides tumor-cell source and PLA2G3-specific loss-of-function linked to lipid-droplet biology, metastasis and platinum sensitization, although spatial niche annotation is limited^[21]^. In Epstein-Barr virus-associated B-cell lymphoma, macrophage-associated PLA2G10 is localized to an extracellular-vesicle niche, hydrolyzes EV phospholipids to generate fatty acids, lysophospholipids, and lipid mediators, and promotes lymphoma growth^[22]^. In other tumor models, PLA2G10-mediated lipid hydrolysis impairs T-cell infiltration and confers resistance to anti-PD-1 therapy, providing a comparatively direct source-lipid-output-immune-function-therapy chain^[23]^. PLA2G1B has a well-defined pancreatic-acinar source and intestinal/systemic lipid-output biology, but its cancer-relevant evidence remains less directly compartment-resolved^[24]^.

These comparisons show that context dependence, lipid-output specificity, and therapeutic redundancy are family-level rather than unique properties of PLA2G2A. We therefore do not claim PLA2G2A to be structurally unique among sPLA2 isoforms. Its relevance to the present review is the combination of ECM retention, M-type receptor/HSPG/integrin/vimentin interactions, and protective intestinal or barrier-associated configurations documented among distinct experimental systems^[3,4,5,7,8,14]^. Direct PLA2G2A-specific evidence for tumor-associated macrophage polarization beyond the ovarian ascites setting, or for tumor-endothelial dysfunction and vascular normalization, remains sparse. Future studies must parallely quantify multiple sPLA2 isoforms and apply the same four-domain annotation to test redundancy, compensation, and isoform-specific druggability.

## MECHANISTIC ROUTES LINKING PLA2G2A TO DRUG RESISTANCE

### Eicosanoid rewiring and chronic inflammatory tone

The most intuitive route from PLA2G2A to therapy resistance is lipid-mediator rewiring. By releasing AA, PLA2G2A can increase substrate availability for COX and LOX enzymes^[1-3]^. COX/PGE2-rich tumor states can impair dendritic-cell function, promote myeloid skewing, restrain cytotoxic T-cell activity and support tumor-promoting inflammation^[25]^. PLA2G2A is unlikely to be the sole determinant of this axis, and the cancer evidence map shows that direct PLA2G2A-linked lipid products are rarely measured in the same specimen or model [Table 2]. Accordingly, lipid-mediated resistance should not be inferred from PLA2G2A expression alone.

**Table 2:** Study-stream, compartment-resolved evidence map for PLA2G2A across cancer settings, separating mechanistic specificity, resistance directness and direct lipid-output evidence

**Cancer context**	**Dominant source/sample**	**Mechanistic/resistance evidence**	**Cancer-biology relevance**	**Therapeutic implication/limitation**	**Key refs**
PDAC	PLA2G2A+ metabolic CAFs	M-A/R2 (single study)^[28]^	PLA2G2A-specific soluble-protein/CAF evidence for CD8^+^ T-cell dysfunction through MAPK/ERK and NF-κB. Lipid-output evidence: no direct AA/PGE2/oxylipin measurement	Prioritize source-resolved CAF and T-cell function biomarkers; direct defined-therapy response and external replication are still required	PDAC CAF^[28]^
Gastric cancer	Tumor epithelium	M-A/R0 for invasion model^[15]^; M-C/R0 for clinical cohorts^[16,17]^	PLA2G2A-specific experimental invasion restraint; favorable clinical associations. Lipid-output evidence: not directly measured	Do not treat epithelial PLA2G2A as uniformly adverse; causal invasion biology is distinct from therapy-resistance evidence	Model^[15]^; clinic^[16,17]^; network^[36]^
Colorectal barrier	Goblet/Paneth epithelial compartments	M-A/M-B/R0 for barrier and microbiota biology^[18,19,30,31]^; M-D for therapy inference	Barrier protection, mucus and antimicrobial defense, microbiota control. Lipid-output evidence: no same-model PLA2G2A-linked AA/PGE2/oxylipin panel	Direct blockade could be harmful in barrier-protective settings; therapy relevance needs disease-stage and mechanism annotation	Mouse^[18,19]^; organoid^[30]^; microbiota^[31]^
Mucinous CRC/EV-active disease	Serum EVs and tumor cells	M-A/R0 for PLA2G2A-specific migration/invasion^[38]^; M-B/R0 for whole-EV pathway data^[38]^	PLA2G2A overexpression increases migration/invasion; Wnt/β-catenin and other pathway changes derive from whole-EV stimulation, not PLA2G2A-specific perturbation. Lipid-output evidence: not directly measured	Keep PLA2G2A-specific function separate from whole-EV pathway enrichment; validate lipids, immune effects and therapy response directly	EV/proteomics/function^[38]^
Peritumoral adipose/ICB MSI-H CRC	Adipose tissue; bulk RNA	M-D/R0 (non-PLA2G2A-specific contextual evidence)^[56,57]^	Peritumoral adipose fibrosis and anti-PD-1 nonresponse provide relevant stromal and immune contexts but do not establish PLA2G2A-specific associations or mechanisms. Lipid-output evidence: not directly linked to PLA2G2A in the same specimen or model	Use as stromal/lipid-context support only; verify PLA2G2A source, spatial location and lipid products directly	Adipose^[56]^; ICB context^[57]^; lipid niche^[34,35]^
Hepatobiliary tumors	GBC epithelium; CCA/HCA tumor tissue	GBC M-A/R0^[39]^; CCA/HCA M-C/R0^[20,40,41]^	GBC overexpression increases proliferation/stemness and reduces apoptosis while reducing migration/invasion in tested models; CCA/HCA evidence is mainly biomarker/epigenetic association. Lipid-output evidence: not directly measured	Interpret phenotype and disease stage separately; no current therapy-resistance evidence	GBC^[39]^; CCA^[20,40]^; HCA^[41]^
OSCC/HNSCC	Invasive-front-associated fibroblasts; inflammatory gene programs	M-B/R0 for OSCC^[32]^; indirect HNSCC treatment-outcome context^[42]^ (not PLA2G2A-specific)	OSCC spatial data identify PLA2G2A-positive fibroblast states near invasive epithelial programs. HNSCC data provide broader inflammatory treatment-outcome associations without establishing a PLA2G2A-specific contribution. Lipid-output evidence: not directly measured	PLA2G2A-specific therapy relevance requires perturbation or dataset-level confirmation	OSCC^[32]^; HNSCC^[42]^
TKI-treated lung cancer	Therapy-stage stroma	M-B/R1 for PLA2G2A-positive CAF treatment-stage association^[33]^	PLA2G2A-positive CAFs were enriched during partial response, whereas POSTN-positive CAFs and ACKR1-positive endothelial cells characterized progressive disease. These findings indicate treatment-stage-associated stromal remodeling rather than PLA2G2A-specific resistance causality. Lipid-output evidence: not directly measured	Possible longitudinal remodeling marker only after treatment-stage confirmation	TKI remodeling^[33]^
Lung squamous cell carcinoma	Immune-related transcriptomes	M-C/R0^[43]^	Reduced PLA2G2A may accompany a colder TME. Lipid-output evidence: not directly measured	Directionality should not be overinterpreted; not a therapeutic target claim	LUSC^[43]^
Prostate cancer	Endocrine-sensitive tumor context; hGIIA-EGFR/vimentin interaction models	M-A/R0 for preclinical PPI targeting^[9]^; M-C/R0 for endocrine, genetic and prognostic associations^[44-47]^	Non-catalytic EGFR/vimentin interactions can be pharmacologically separated from catalytic function in preclinical models. Lipid-output evidence: not the primary readout	Use as a mechanistic model for non-catalytic targeting, not as clinically validated resistance evidence	Endocrine^[44]^; signatures^[45,46]^; variant^[47]^; PPI inhibitor^[9]^
Renal/bladder/endometrial cancer	Prognostic, age-related and immune/lipid signatures	M-C/R0 for renal prognostic and age-related signatures^[48,49]^; M-C/R1 for predicted treatment-response signatures^[51-53]^	ccRCC Ref.^[48]^ is an immunogenomic prognostic model; Ref.^[49]^ is an age-related DEG/prognostic analysis. Bladder/endometrial studies are signature-based. Lipid-output evidence: not directly measured	Hypothesis-generating only; avoid standalone mechanism or target claims	ccRCC immune^[48]^; ccRCC age^[49]^; redox context^[50]^; bladder^[51]^; endometrial^[52,53]^
Breast/ovarian cancer	PLA2G2A+ CAFs or ascites lipid milieu	Breast M-B/R0 for CAF profiling^[54]^; M-A/R0 for cancer-cell functional evidence^[55]^; ovarian M-B/R0^[29]^	Breast data show compartment divergence. Ovarian ascites links extracellular PLA2G2A to AA and AA to macrophage stress/vesicle release; the full chain lacks PLA2G2A-specific perturbation. Lipid-output evidence: ovarian AA measured; vesicles not shown to be AA-rich	Separate tumor-cell, CAF and fluid-phase PLA2G2A; ovarian therapy inference requires direct perturbation	Ovarian^[29]^; breast^[54,55]^

Evidence grades were assigned according to the review-specific dual-axis framework described in Table 1. References are provided for each evidence classification. PLA2G2A-specific functional evidence is distinguished from treatment-stage associations, multigene signatures, and indirect contextual evidence. The assigned grades reflect the evidence supporting the specific claim rather than the overall quality of the cited studies. PDAC: Pancreatic ductal adenocarcinoma; CAFs: cancer-associated fibroblasts; M: mechanistic specificity; R: resistance directness; MAPK: mitogen-activated protein kinase; ERK: extracellular signal-regulated kinase; NF-κB: nuclear factor κB; AA: arachidonic acid; PGE2: prostaglandin E2; CRC: colorectal cancer; EVs: extracellular vesicles; ICB: immune-checkpoint blockade; MSI-H: microsatellite instability-high; PD-1: programmed cell death protein 1; GBC: gallbladder carcinoma; CCA: cholangiocarcinoma; HCA: hepatocellular adenoma; OSCC: oral squamous-cell carcinoma; HNSCC: head and neck squamous-cell carcinoma; TKI: tyrosine kinase inhibitor; POSTN: periostin; ACKR1: atypical chemokine receptor 1; TME: tumor microenvironment; LUSC: lung squamous cell carcinoma; hGIIA: human group IIA secreted phospholipase A_2_; EGFR: epidermal growth factor receptor; PPI: protein-protein interaction; ccRCC: clear cell renal cell carcinoma; DEG: differentially expressed gene.

For therapeutic design, the distinction between upstream enzyme expression and downstream lipid output is essential. If PLA2G2A mainly supplies substrate to a COX/PGE2-dominant niche, downstream lipid-pathway blockade may be more rational than direct PLA2G2A inhibition. If its dominant role is receptor or matrix coupling, catalytic inhibition alone may fail. Therapy-oriented studies should therefore pair source-resolved PLA2G2A measurements with direct lipidomics when a lipid mechanism is proposed, receptor or ECM markers when non-catalytic biology is proposed, and immune-functional endpoints [Figure 2].

**Figure 2:**
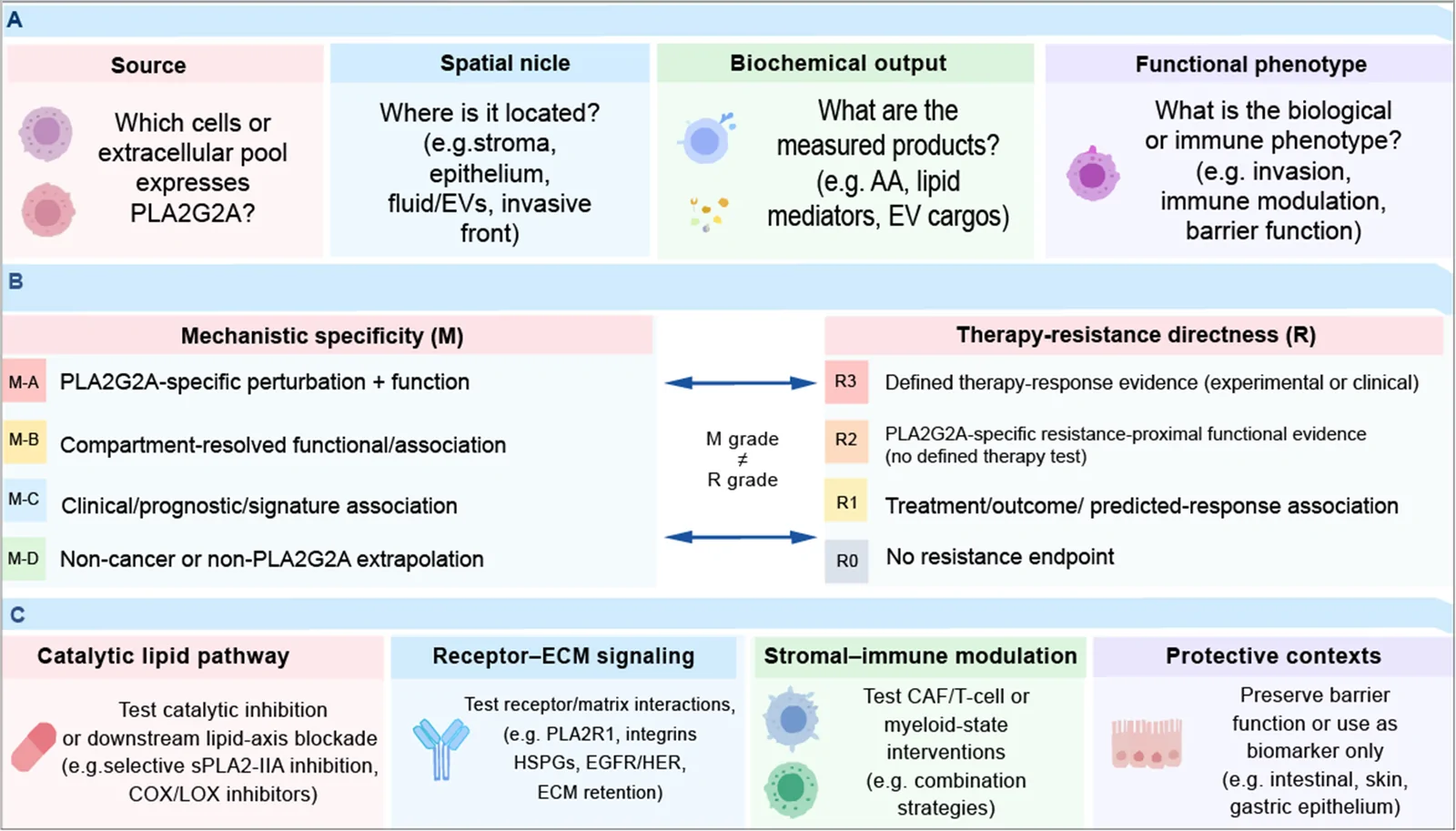
Evidence-gated interpretation and mechanism-matched therapeutic testing for PLA2G2A. (A) Compartment annotation: four essential domains; (B) Two orthogonal evidence axes(not hierarchical); (C) Mechanism-matched therapeutic strategies (hypothesis-driven). EVs: Extracellular vesicles; AA: arachidonic acid; sPLA2-IIA: group IIA secreted phospholipase A2; COX: cyclooxygenase; LOX: lipoxygenase; ECM: extracellular matrix; PLA2R1: phospholipase A2 receptor 1; HSPGs: heparan sulphate proteoglycans; EGFR: epidermal growth factor receptor; HER: human epidermal growth factor receptor; CAF: cancer-associated fibroblast.

### Fibroblast-mediated T-cell dysfunction

The strongest resistance-proximal PLA2G2A-specific evidence comes from fibroblast-rich PDAC. CAFs regulate ECM architecture, cytokine gradients, immune-cell trafficking, and treatment resistance^[26,27]^. In PDAC, PLA2G2A-positive metabolic CAFs suppress CD8^+^ T-cell antitumor activity, reduce IFN-γ and granzyme B secretion, and impair cytotoxic function as well as MAPK/extracellular signal-regulated kinase (ERK) and NF-κB signaling in T cells^[28]^. As this study demonstrates PLA2G2A-specific immune dysfunction without testing the outcome to a defined anticancer therapy, it is classified as M-A/R2 rather than as direct therapy-response evidence. The therapeutic point is that CD8^+^ T cells may remain but are functionally disabled; it differs from simple lymphocyte exclusion and implies that treatments aimed only at T-cell recruitment may be insufficient. A rational preclinical strategy would combine CAF-state modulation, recovery of T-cell effector signaling and checkpoint blockade, with spatial CAF-CD8 proximity and T-cell functional markers as pharmacodynamic readouts.

### Myeloid remodeling and secretory lipid niches

Ovarian cancer illustrates a fluid-phase lipid niche. Ovarian-cancer ascites contain abundant AA, and phosphoproteomics identified PLA2G2A as a clinically relevant extracellular enzyme associated with this pool^[29]^. In macrophages, AA activated Ca^2+^/CaMKII-ASK1-p38 signaling, induced a stress program associated with poor outcome, impaired actin-driven macropinocytosis, and enhanced release of tetraspanin-containing vesicles^[29]^. The study therefore supports AA-induced vesicle release; it does not establish that these vesicles are themselves AA-rich. Such evidence should be framed as a mechanistic association across linked but not fully PLA2G2A-perturbed steps rather than definitive proof of PLA2G2A alone driving ovarian immune escape. Direct PLA2G2A perturbation across the complete pathway is imperative. Nonetheless, the model suggests that ascite PLA2G2A activity, arachidonic-acid abundance, macrophage stress programs, and vesicle-release phenotypes could be co-evaluated in therapy-response studies.

### Barrier dysfunction, microbiota and distal immune conditioning

Barrier tissues provide the clearest example of opposing PLA2G2A outputs. In the homeostatic intestine, PLA2G2A can contribute to mucosal protection and antimicrobial or microbial-defense programs^[18,19]^. During chronic inflammatory injury, however, the same barrier-associated program may coexist with dysbiosis, cytokine exposure and tissue repair signals that condition future tumor immunity.

Human colon organoids exposed to pro-inflammatory conditions show increased PLA2G2A together with impaired barrier programs, whereas a multi-mineral intervention reduces PLA2G2A and improves epithelial cohesion and transepithelial resistance^[30]^. Intestinal sPLA2-IIA can also reshape distant skin carcinogenesis by modifying the gut microbiota^[31]^. These findings argue against default systemic PLA2G2A blockade in barrier organs; in selected settings, barrier repair or microbiota-aware intervention may be more rational.

### Therapy-induced plasticity and invasive-front ecology

Therapy and invasion expose cell states that may be missed by treatment-naive bulk profiling. In oral squamous-cell carcinoma (OSCC), single-cell and spatial multi-omics identified an ADH1B/MFAP4/PLA2G2A fibroblast subgroup positioned near invasive epithelial programs characterized by copy-number instability, glycolysis, hypoxia, reactive-oxygen-species adaptation and epithelial-mesenchymal transition^[32]^. This suggests that PLA2G2A-positive fibroblasts may mark a boundary-zone stromal state where tissue repair, oxidative stress and invasive selection pressure converge.

Single-cell analyses of tyrosine kinase inhibitor (TKI)-treated lung cancer reveal stage-dependent stromal remodeling, with resistant lesions dominated by periostin (POSTN)-positive CAFs and ACKR1-positive endothelial cells^[33]^. Any PLA2G2A-related CAF signal in this setting should therefore be interpreted as a candidate transitional feature rather than as a validated recurrent resistance marker. Longitudinal sampling is essential to distinguish adaptive remodeling from stable resistance architecture.

### Adipocyte, extracellular-vesicle and lipid-niche communication

A broader lipid-niche model is needed because PLA2G2A acts in environments where extracellular lipid availability, adipocyte biology, inflammation and vesicular communication intersect. Reviews of cancer lipid metabolism emphasize that tumor lipid states are shaped by cell-extrinsic nutrients, adipocytes, stromal cells, hypoxia and physical TME features, and that lipid availability can propagate metabolic ripple effects beyond canonical lipid pathways^[34,35]^. In this setting, PLA2G2A is best viewed as a potential amplifier of a lipid-rich inflammatory niche rather than as a sole upstream driver.

Adipocytes and adipose-associated stromal or immune cells can supply fatty acids, inflammatory cytokines and EVs, while EVs can package enzymes, lipids and matrix-interacting proteins. PLA2G2A in this context may indicate a secretory lipid-processing state that couples adipose inflammation, myeloid remodeling, mucin production or ECM remodeling. The interpretation remains hypothesis-generating unless PLA2G2A is spatially localized, lipid products are quantified and perturbation changes EV composition, macrophage state, CAF behavior or treatment response.

## CANCER-CONTEXT EVIDENCE

### Gastrointestinal and hepatobiliary tumors

Gastrointestinal tumors demonstrate why PLA2G2A must be interpreted by compartment, phenotype, and evidence axis. In gastric cancer experimental models, epithelial PLA2G2A suppresses invasion and metastasis^[15]^, providing M-A evidence for an invasion-restraining phenotype, while patient cohorts associate PLA2G2A expression with favorable clinicopathologic features and survival^[16,17]^, which remains M-C clinical evidence. A gastric-cancer coexpression meta-network places PLA2G2A within a broader epithelial program^[36]^. None of these studies directly establish therapy resistance (R0). CRC contains both protective and tumor-supportive configurations. In mouse models, PLA2G2A can protect against colorectal tumorigenesis when integrated with mucus and antimicrobial programs^[18,19]^. In sporadic colorectal adenocarcinoma, bulk PLA2G2A expression is not uniformly enhanced compared with normal mucosa^[37]^, implying that average tissue expression may miss disease states in which PLA2G2A gains therapeutic relevance.

Mucinous colon adenocarcinoma illustrates why PLA2G2A-specific perturbation and whole-EV pathway evidence must be separated. Serum-derived EV proteomics demonstrated PLA2G2A enrichment in mucinous compared with nonmucinous tumors^[38]^. In a separate PLA2G2A-specific experiment, PLA2G2A overexpression in SW480 cells enhanced migration and invasion. By contrast, Wnt/β-catenin and other changes in cancer-related pathways were derived from recipient cell proteomics after stimulation with whole mucinous-cancer EVs and therefore cannot be attributed specifically to PLA2G2A^[38]^. Any direct PLA2G2A-linked AA, PGE2, oxylipin, or lysophospholipid measurement was not reported in that model. These data support M-A evidence for migration/invasion and M-B proof for the EV compartment, but R0 for direct treatment resistance.

Hepatobiliary evidence is heterogeneous. Gallbladder-carcinoma single-cell analysis identified PLA2G2A-positive epithelial states and included PLA2G2A overexpression showing elevated proliferation and stemness, reduced apoptosis and, crucially, declined migration/invasion within the tested cell models^[39]^, constituting M-A functional but not therapy-response evidence (R0). PLA2G2A has been proposed as a diagnostic or prognostic biomarker for cholangiocarcinoma^[40]^, whereas epigenetic data indicate that PLA2G2A repression may accompany progression within certain contexts^[20]^. In inflammatory hepatocellular adenoma, PLA2G2A marks an inflammation-rich subtype^[41]^. These divergent observations support stage- and phenotype-dependent interpretation rather than a static tumor-promoting model.

### Pancreatic and squamous tumors

PDAC is currently the most compelling setting for a causal stromal immune-dysfunction model. PLA2G2A-positive metabolic CAFs suppress CD8^+^ T-cell effector function through MAPK/ERK and NF-κB inhibition, reduce effector cytokines and blunt tumor killing^[28]^. This places PLA2G2A within a stromal immune-escape program rather than a tumor-cell-autonomous oncogene. Under the revised framework, the study is M-A/R2: PLA2G2A-specific functional evidence is strong, but a defined anticancer therapy-response experiment was not performed. Independent cohorts, orthogonal spatial proteomics, lipidomic readouts and immunocompetent therapy-response models are needed before extrapolation to other tumors.

Squamous tumors add spatial and therapy-stage nuance. In OSCC, PLA2G2A-positive fibroblasts may identify an invasive-front branch point where tissue-preserving stromal functions are co-opted into tumor-supportive ecology^[32]^. In recurrent or metastatic head and neck squamous-cell carcinoma (HNSCC) treated with dacomitinib, exploratory biomarker analysis linked high inflammatory gene-expression signals, particularly cytokine-centered programs, with shorter progression-free survival^[42]^. These observations are compatible with a resistance-associated inflammatory niche, but they do not establish PLA2G2A-specific druggability.

### Lung and genitourinary tumors

In lung squamous-cell carcinoma, loss of representative tumor-suppressor genes was associated with a colder microenvironment and reduced expression of immune-system-related genes including PLA2G2A^[43]^. Thus, diminished PLA2G2A is not automatically favorable; in specific squamous ecosystems, it may accompany the collapse of productive immune-stromal crosstalk. Both high- and low-intensity PLA2G2A signals therefore require context-resolved interpretation. In prostate cancer, PLA2G2A must be treated as a limited-evidence-value, endocrine-sensitive signal rather than a validated stromal driver. Cell-line data suggest that 5α-reductase suppression can alter gene-expression programs including *PLA2G2A*^[44]^. Prognostic and immune-infiltration analyses associate PLA2G2A-containing signatures with an immune context and outcome^[45,46]^; a 5′ UTR polymorphism is linked to metastatic risk^[47]^. Preclinical protein-protein interaction inhibitors provide a mechanistic model for noncatalytic targeting^[9]^, but clinical applicability remains unresolved.

Renal and bladder cancers provide mostly correlative evidence. In clear-cell renal-cell carcinoma, one immunogenomic analysis constructed an immune-related prognostic model that included *PLA2G2A* among prognostic immune-system-related genes^[48]^. A separate TCGA/SEER study addressed another question - age stratification and age-related differentially expressed genes - and identified *PLA2G2A* as an age-associated gene linked to poorer prognosis in older patients rather than as an immune-risk model^[49]^. Separate studies on ferroptosis and glutathione indicate that redox adaptation is relevant in ccRCC, but these data are not PLA2G2A-specific^[50]^. In bladder cancer, PLA2G2A is incorporated into lipid-metabolism signatures associated with prognosis, immune infiltration, and predicted immunotherapy response^[51]^. These reports support stratification hypotheses but do not justify PLA2G2A as an independent therapeutic target.

### Reproductive-system tumors

Endometrial cancer studies repeatedly place PLA2G2A within immune-risk classifiers that stratify survival, tumor mutation burden, immune infiltration, and predicted response to immuno- or chemotherapy^[52,53]^. The signal appears to reflect immune-state organization rather than uniform tumor-cell overexpression. These signatures may be clinically applicable for risk stratification, but cannot distinguish whether PLA2G2A is a driver, an inflammatory passenger, or a surrogate for immune-cell composition. Breast cancer underscores compartmental divergence. Single-cell profiling of primary tumors and paired lymph-node metastases identified a PLA2G2A-positive CAF subset enriched in HER2-positive disease and interacting with T cells, dendritic cells, and macrophages^[54]^. By contrast, experimental breast-cancer models implicate a PLA2G2A/DCN axis in stachydrine-associated growth restraint^[55]^, reinforcing the core principle that epithelial and stromal PLA2G2A should not be merged for biological relevance.

Ovarian cancer represents a secretory lipid-milieu configuration. Ascites-derived AA is clinically adverse, and PLA2G2A is implicated as an extracellular enzyme contributing to this lipid pool^[29]^. The downstream macrophage findings are AA-driven, including stress signaling, impaired macropinocytosis, and AA-induced release of tetraspanin-containing vesicles; the study does not show that those vesicles are AA-enriched. Direct PLA2G2A perturbation, paired ascites lipidomics, and macrophage-state readouts are needed to move this setting from compartment-linked association toward therapeutic causality.

## BIOMARKER AND THERAPEUTIC IMPLICATIONS

### Compartment-resolved biomarkers

Bulk RNA or serum PLA2G2A assessments alone are unlikely to be clinically interpretable. A therapy-relevant biomarker should distinguish epithelial *vs.* stromal source, tissue-retained *vs.* circulating or EV-associated pools, tumor core *vs.* invasive-front localization, and treatment-naive *vs.* therapy-remodeled states. The study-level evidence and the occurrence of direct lipid-output measurements are summarized in Table 2. Without these distinctions, PLA2G2A may appear inconsistent across studies as assays measure varied biological compartments and evidence domains.

A minimum prospective biomarker panel must include PLA2G2A source compartment, CAF or epithelial markers, and a mechanism-matched output. When a catalytic lipid mechanism is proposed, it has to include direct AA/PGE2/oxylipin or lysophospholipid assessments rather than inferred pathway activity. When an immune mechanism is proposed, markers such as IFNG, GZMB, CD69, and OX40 in CD8^+^ T cells and relevant myeloid stress markers must be quantified. Longitudinal studies have to sample the same markers before treatment, during response, and at progression to determine whether PLA2G2A marks a transient remodeling window or a stable resistance state.

### Mechanism-matched therapeutic hypotheses

The evidence argues against a one-size-fits-all PLA2G2A inhibitor strategy. In a catalytic lipid-dominant context, downstream COX/PGE2 or LOX-axis blockade may be more rational than direct PLA2G2A inhibition. In a CAF-dominant context, fibroblast-state modulation plus T-cell reactivation may be worth testing as a preclinical combination hypothesis. In a secretory lipid niche such as ovarian ascites, macrophage-state reprogramming and lipid-axis blockade require direct validation. In barrier organs, restoring epithelial integrity or microbiota balance may be preferable to direct PLA2G2A blockade. These scenarios should be treated as mechanism-matched trial-enabling hypotheses, not as immediate clinical recommendations.

Mechanistic matching is especially important because sPLA2-IIA can signal through M-type receptor and HSPG-dependent amplification^[4,5]^, direct integrin binding^[7]^, EGFR/HER-family or vimentin-associated interactions^[6,8,9]^, and matrix retention^[14]^. A prognostic association does not justify targeting unless perturbation changes the relevant immune, lipid, stromal or therapy-response phenotype. Peritumoral adipose fibrosis and microsatellite instability-high (MSI-H)/mismatch-repair deficient (dMMR) anti-PD-1 nonresponse provide additional TME contexts in which the potential contribution of PLA2G2A should be investigated directly rather than assumed^[56,57]^.

### Inhibitor feasibility and safety

A major translational bottleneck is the absence of a clinically approved PLA2G2A-specific anticancer inhibitor. Varespladib/varespladib methyl inhibits multiple secreted PLA2 isoforms^[3]^ and has been clinically evaluated outside oncology; in acute coronary syndrome, the VISTA-16 randomized trial did not support benefit and raised safety concerns^[58]^. Systemic catalytic blockade therefore cannot be proposed as a straightforward cancer strategy without patient selection, pharmacodynamic monitoring and tissue-specific safety assessment.

Non-catalytic targeting remains clinically immature. Cyclic peptides that interfere with hGIIA-EGFR and hGIIA-vimentin interactions provide preclinical proof-of-principle^[9]^, but their applicability outside prostate cancer is unknown. Compartment-restricted strategies such as local delivery, ascites-directed intervention, CAF-targeted carriers, ECM-activated prodrugs or EV-associated capture remain experimental concepts. Validation should include enzyme activity, protein-protein binding, spatial pharmacodynamics, lipidomics, immune readouts and barrier-toxicity assays.

### Research priorities

The field now needs fewer bulk-expression catalogs and more mechanism-matched validation. Key questions include which PLA2G2A-positive cell states are functionally immunosuppressive, which are protective or reactive, which outputs depend on catalytic lipid release, and which depend on receptor or matrix coupling. It is also essential to test whether spatial biomarkers can identify patients whose tumors are truly PLA2G2A-dependent and whether PLA2G2A marks an early remodeling window that can be interrupted before terminal resistance architecture develops. These priorities are summarized with study-specific evidence codes and citations in Table 3.

**Table 3:** Evidence-gated, therapy-oriented prioritization matrix for PLA2G2A-directed studies. Relevant literature is cited within each evidence-status cell

**Clinical/ecological setting**	**Evidence status**	**Mechanism-matched hypothesis**	**Required validation/translational bottleneck**
PDAC with PLA2G2A+ CAF enrichment	M-A/R2, single-study immune-functional evidence^[28]^	Test CAF-state modulation with T-cell reactivation or checkpoint therapy as a preclinical combination hypothesis	PLA2G2A+ CAF density; spatial CAF-CD8 proximity; CD8 IFNG/TNF/GZMB/CD69/OX40; MAPK/NF-κB activity; defined therapy-response experiment; independent validation
Mucinous CRC with EV-active phenotype	M-A/R0 for PLA2G2A migration/invasion; M-B/R0 for whole-EV pathway data^[38]^	Pair EV/niche studies with mechanism-matched lipid or stromal intervention only after direct biochemical and immune validation	EV-PLA2G2A; BRAF status; AA/PGE2/oxylipins as prespecified lipid outputs; separate whole-EV pathway scores; PLA2G2A-specific perturbation
Ovarian cancer ascites	M-B/R0 secretory lipid-niche evidence^[29]^	Test PLA2G2A-specific perturbation before combining lipid-axis blockade with macrophage-state reprogramming	Ascites AA; PLA2G2A abundance/activity; CaMKII-ASK1-p38 signaling; AA-induced vesicle release; direct PLA2G2A inhibition or knockdown
Barrier-disrupted intestinal lesions	M-A/M-B/R0 for barrier biology; M-D for cancer-therapy inference^[18,19,30,31]^	Prioritize barrier repair or microbiota-aware strategies before considering direct blockade	Mucus integrity; epithelial PLA2G2A; inflammatory cytokines; microbiota composition; mechanism-matched lipid readouts if claimed; epithelial toxicity and dysbiosis risk
Therapy-remodeled lung or squamous tumors	M-B/M-C/M-D; R0-R1 depending on dataset^[32,33,42]^	Treat PLA2G2A as a candidate remodeling marker only after treatment-stage and PLA2G2A-specific confirmation	PLA2G2A if detectable; POSTN CAFs; ACKR1 endothelial cells; inflammatory programs; longitudinal treatment-stage sampling; direct perturbation

PDAC: Pancreatic ductal adenocarcinoma; CAF: cancer-associated fibroblast; M: mechanistic specificity; R: resistance directness; IFNG: interferon gamma; TNF: tumor necrosis factor; GZMB: granzyme B; MAPK: mitogen-activated protein kinase; NF-κB: nuclear factor kappa B; CRC: colorectal cancer; EV: extracellular vesicle; BRAF: B-Raf proto-oncogene, serine/threonine kinase; AA: arachidonic acid; PGE2: prostaglandin E2; CaMKII: calcium/calmodulin-dependent protein kinase II; ASK1: apoptosis signal-regulating kinase 1; POSTN: periostin; ACKR1: atypical chemokine receptor 1.

## LIMITATIONS AND UNRESOLVED QUESTIONS

Several limitations should be made explicit. First, this review is a narrative synthesis and does not include original data mining, formal meta-analysis, or spatial-transcriptomic reanalysis. Second, the evidence map contains more PLA2G2A-specific functional cancer biology than the original single-axis grading implied, but direct treatment-response evidence remains sparse: most studies are R0-R1, and the PDAC CAF report is R2 rather than R3. Third, grade, stage, differentiation state, immune infiltration, stromal abundance, and lipid-metabolism programs are major confounders that are not consistently controlled across studies. Fourth, PLA2G2A-linked lipid products are rarely quantified in the same specimens or models for which source and localization were established. Fifth, mechanisms inferred from noncancer inflammatory, vascular, adipose, or barrier models may not operate identically in malignant tissues, and sPLA2-family redundancy and compensatory lipid pathways are rarely measured in parallel. Finally, clinical translation is constrained by a lack of validated PLA2G2A-selective anticancer inhibitors, uncertainty about noncatalytic targeting, and possible safety concerns within antimicrobial, vascular, and epithelial-barrier contexts.

These limitations do not negate the value of a compartment-resolved framework; they restrict the current claims. PLA2G2A has to be regarded as a conditional biomarker and candidate pathway whose mechanistic specificity and therapy-response relevance must be scored separately. Therapeutic conclusions require source-resolved localization, a directly assessed mechanism-matched output and perturbational validation within the relevant treatment context.

## CONCLUSIONS

PLA2G2A lies at the convergence of lipid metabolism, extracellular signaling, stromal ecology and barrier biology. Its biologically relevant meaning is therefore compartment-dependent rather than uniformly tumor-promoting or protective. PLA2G2A-specific functional evidence exists for several epithelial and stromal cancer models, but direct evidence of PLA2G2A changing the response to a defined anticancer therapy remains limited. The most robust current resistance-proximal setting was PLA2G2A-positive PDAC CAF-mediated CD8^+^ T-cell dysfunction, whereas ovarian ascites, mucinous colorectal EVs, therapy-remodeled stroma, and clinical signatures remain incompletely mechanistic or treatment-responsive chains. Conversely, gastric and intestinal epithelial contexts illustrate invasion-restraining or barrier-protective biology that could render indiscriminate systemic blockade undesirable. Future translation should therefore require the convergence of source, niche, mechanism-matched biochemical output, functional dependence, and therapy-response evidence. In practical terms, PLA2G2A is best treated as a context-qualified biomarker and therapeutic hypothesis, not as a universal target.

## DECLARATIONS

### AI and AI-assisted tools statement

During manuscript preparation and revision, the authors used ChatGPT (GPT-5.6 Sol, OpenAI; accessed 24 August 2026) only to improve sentence structure and language clarity. All AI-assisted output was reviewed, verified and edited by the authors, who take full responsibility for the final content. No AI-generated experimental data, analyses or references were used.

### Copyright

© The Author(s) 2026.
